# ODNet: A High Real-Time Network Using Orthogonal Decomposition for Few-Shot Strip Steel Surface Defect Classification

**DOI:** 10.3390/s24144630

**Published:** 2024-07-17

**Authors:** He Zhang, Han Liu, Runyuan Guo, Lili Liang, Qing Liu, Wenlu Ma

**Affiliations:** 1 School of Automation and Information Engineering, Xi’an University of Technology, Xi’an 710048, China; hezhang@stu.xaut.edu.cn (H.Z.); guorunyuan@xaut.edu.cn (R.G.); llliang@xaut.edu.cn (L.L.); itcliuq@xaut.edu.cn (Q.L.); 2 School of Information Engineering, Shannxi Xueqian Normal University, Xi’an 710100, China; 28030@snsy.edu.cn

**Keywords:** real time, orthogonal decomposition, skip connection, few-shot defect classification, Euclidean distance

## Abstract

Strip steel plays a crucial role in modern industrial production, where enhancing the accuracy and real-time capabilities of surface defect classification is essential. However, acquiring and annotating defect samples for training deep learning models are challenging, further complicated by the presence of redundant information in these samples. These issues hinder the classification of strip steel surface defects. To address these challenges, this paper introduces a high real-time network, ODNet (Orthogonal Decomposition Network), designed for few-shot strip steel surface defect classification. ODNet utilizes ResNet as its backbone and incorporates orthogonal decomposition technology to reduce the feature redundancies. Furthermore, it integrates skip connection to preserve essential correlation information in the samples, preventing excessive elimination. The model optimizes the parameter efficiency by employing Euclidean distance as the classifier. The orthogonal decomposition not only helps reduce redundant image information but also ensures compatibility with the Euclidean distance requirement for orthogonal input. Extensive experiments conducted on the FSC-20 benchmark demonstrate that ODNet achieves superior real-time performance, accuracy, and generalization compared to alternative methods, effectively addressing the challenges of few-shot strip steel surface defect classification.

## 1. Introduction

In the industrial age, the demand for strip steel across various industries is increasing. However, due to the influence of temperature and manufacturing processes, surface defects such as water spots, creases, and patches frequently occur during production. These defects can seriously affect both the quality and safety of the final product [1,2]. If such defects are not identified in a timely manner, they can lead to significant losses in subsequent production stages. Therefore, it is crucial to quickly and accurately classify surface defects in strip steel production [3,4].

With the rise of deep learning, industrial production has transitioned from traditional to intelligent manufacturing, infusing artificial intelligence with new vigor [5,6,7]. Many researchers have applied deep learning techniques to classify strip steel surface defects. The fusion matrix based on Fisher’s criterion and correlation analysis was introduced in [8], effectively integrating global and local dimensions. Classification performance was improved using multi-label techniques in [9], with model complexity and latency for small datasets reduced. Consideration of the correlation between pixel-level segmentation masks, object-level bounding boxes, and global image-level classification labels was undertaken in [10], with the joint learning of the features of related tasks to improve the performance. A scheme based on ResNet50 with FcaNet and Convolutional Block Attention Module (CBAM) for strip defect classification was proposed in [11]. The CutPaste-Mix data augmentation strategy and Gaussian Density Estimation for abnormal region classification were utilized in [12].

However, in actual industrial production, surface defects on strip steel are rare and challenging to acquire. Therefore, directly applying traditional deep learning methods to classify these defects often leads to overfitting issues [13,14]. Moreover, industrial images contain significant redundant information, further complicating the task of classification [15,16,17].

Inspired by the human ability to quickly learn from a small number of examples, few-shot learning emerged [18]. Its goal is to train a classifier using a limited number of samples that can then efficiently detect new defects with a small number of samples [19,20]. This necessitates high precision and robust generalization from the model, aligning more closely with the practical demands of industrial defect classification [21].

The few-shot strip steel surface defect classification model is mainly divided into three methods: data augmentation-based, optimization-based, and metric-based [22,23].

**Data augmentation-based**. This is the most direct approach to addressing the few-shot strip steel surface defect classification problem, which can be extended through affine transformations such as rotation, cropping, or online enhancements like Generative Adversarial Networks (GAN) [24] and CutMix [25,26,27]. Data augmentation methods such as those proposed in [28] involve accumulating richly featured data incorporating expert knowledge of abnormalities, including diverse features, positions, sizes, and backgrounds. The residual discriminator network structure within a dual discriminator GAN framework was introduced in [29] to enhance generation diversity while preserving image features. Recognition generalization across meta-tasks is improved by a meta-augmentation method proposed in [30] through joint parameter updating from original and augmented domains.

**Optimization-based**. Gradient optimization enables rapid adaptation to new tasks [31,32]. MAML [33] is recognized as one of the most influential methods, with iterative models updated by amalgamating gradients, thereby influencing numerous subsequent methodologies. A hyperparametric adaptive strategy based on gradient descent (HASGD) is introduced in [34] to enhance the stability and scalability of the training process. The framework and neural network models are refined in [35] based on MAML [33].

**Metric-based**. This is one of the most common solutions for few-shot strip steel surface defect classification, primarily comprising a classifier and feature extractor, which categorize samples by mapping nonlinear maps in the embedding space [36,37,38]. A novel dual-stream neural network is proposed, involving the generation of numerous defect samples for classifier pretraining, and the classification of real steel strip surface defects is achieved using the transfer learning method [39]. A transductive learning algorithm was designed and presented in [40], where a new classifier was trained during the test phase to accommodate the needs of unknown samples. A depth metric-based classification method is proposed in [41] to identify a sample-matching feature space with effective similarity measures using cosine distance. A transductive few-shot surface defect classification method is introduced in [42], leveraging both instance-level and distribution-level relations within each few-shot learning task. ResMSNet, a novel backbone network presented in [43], draws on the idea of multi-scale feature extraction for small discriminative regions in defect samples and provides classification via linking prototype distances and nonlinear relation scores. CPANet, proposed in [44], effectively aggregates long-range relationships of discrete defects and introduces a space squeeze attention module to aggregate multiscale context information of defect features. An attention-guided recognition network is presented in [45], featuring channel and position attention modules and a dual-metric function for learning classification boundaries by controlling sample distances in the feature space between intraclass and interclass. Benefiting from the simplicity, high efficiency, and strong designability of metric-based methods, the model proposed in this work also falls into the category of metric-based approaches.

With the advances in deep learning technology, mainstream models are becoming increasingly complex. However, as the model complexity grows, real-time performance is adversely affected, which fails to meet the demands of industrial production. Conversely, simpler models lack the capability to extract intricate discriminative features, thereby compromising classification performance. Moreover, strip surface defects typically occupy a small portion of the overall image, with the majority consisting of redundant information. Addressing the issue of few-shot learning, the model’s effectiveness is hindered by insufficient sample data, necessitating the minimization of redundant information interference to enhance the model’s utilization of pertinent data.

Before the widespread adoption of deep learning, earlier studies utilized Singular Value Decomposition (SVD) to address redundancy in the classification of strip surface defects. Ref. [46] presents a technique for the detection of local defects in cold rolled strips. In their approach, principal component analysis is employed with SVD to reduce the dimensionality of the extracted feature vector. Subsequently, the defects in the steel strips are detected using a feed-forward neural network. An approach is proposed in [47], where the gray level matrix of a digital image is projected onto its singular vectors obtained through SVD. Defects are identified by abrupt changes in these projections, allowing for the determination and rough localization of the defects. The effectiveness of traditional machine learning also provides inspiration for this work. The combination of traditional methods with deep learning can yield improved results.

To tackle the above challenges, this study introduces ODNet, a high real-time network that utilizes orthogonal methods to mitigate the influence of redundant information on the model and maximize the utility of the limited available data. ODNet achieves de-redundancy via the orthogonal decomposition of fully connected layer parameters, ensuring orthogonal feature projection. The model incorporates hops to safeguard against the loss of useful information during orthogonal decomposition operations. This orthogonal embedding of features enhances its suitability for Euclidean distance inputs. Experiments were conducted on the FSC-20 benchmark, specifically designed to validate the few-shot strip steel surface defect classification model. ODNet demonstrates superior classification accuracy, high real-time performance, and strong generalization compared to other methods. Additionally, extensive ablation experiments were conducted to assess the influence of the model parameters and modules on the performance.

Accordingly, this paper makes the following four major contributions:

- A high real-time network for few-shot strip steel surface defect classification is proposed.
- ODNet employs orthogonal decomposition to derive orthogonal features, thereby minimizing the impact of redundant information on the model. The inclusion of a skip connection ensures that the valuable correlation information remains intact, especially after orthogonal decomposition.
- The features extracted by the model with orthogonality also adhere more closely to the orthogonality requirement of the Euclidean distance on input, thereby enhancing the classifier performance.
- Compared to alternative methods, ODNet exhibits superior real-time performance, precision, and generalization, aligning more closely with the specific demands of industrial production.

The proposed method is described in detail in Section 2, Section 3 provides the details of a series of experiments to verify the performance of the model. Finally, this paper discusses and summarizes the proposed method in Section 4 and Section 5.

## 2. Methodology

The problem definition of few-shot strip steel surface defect classification is explained in Section 2.1, and the proposed network and its used loss function are detailed in Section 2.2.

### 2.1. Problem Definition

**Figure 1:**
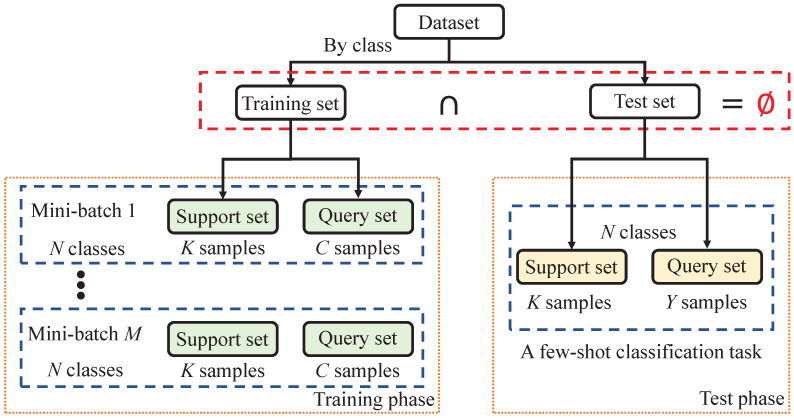
Episodes’ training process. The testing phase presents a few-shot classification task.

Given a dataset *D*, it is divided into a mutually exclusive training set $D_{train}$ and testing set $D_{test}$ by class. Mini-batches are randomly selected from the training set. Each mini-batch includes *N* classes, with *K* samples from each class forming the support set and *C* samples from each class forming the query set. Multiple mini-batches are selected to iteratively update the model. The same steps are repeated during the testing phase. This unique training process is referred to as **episodes**, designed to simulate the few-shot strip steel surface defect classification scenario and to objectively evaluate the model’s performance. Figure 1 illustrates this process.

### 2.2. ODNet

**Figure 2:**
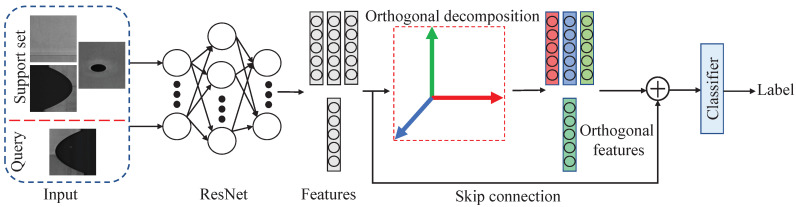
ODNet architecture, where colored boxes represent orthogonal features.

The largest limitation of few-shot steel strip defect classification is that the number of samples is small, the usefulness of the samples is limited, and there is a lot of redundant information. This makes it easy for the model to learn too much redundant information, reducing the impact of the useful information on the model and leading to overfitting. The ODNet proposed in this paper alleviates the negative impact of redundant information on model training through orthogonal decomposition operations, while better meeting the requirement of the Euclidean distance for orthogonal input features and improving the classification performance of the model. As shown in Figure 2, the model uses ResNet [48] as the backbone to perform orthogonal decomposition on the input features, while adding a skip connection to ensure that the orthogonal decomposition operation does not erroneously filter out useful information in the samples. Finally, a classifier is used to obtain the predicted labels.

#### 2.2.1. Feature Extractor

**Figure 3:**
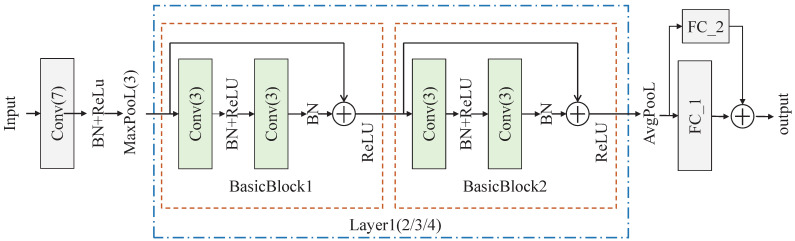
The pipeline of the feature extractor. Layer1, Layer2, Layer3, and Layer4 have the same structure.

ODNet utilizes ResNet18 as the backbone feature extractor, known for its ability to effectively extract deep sample features while mitigating overfitting. The feature $f_{\phi }\left(x\right)$ is derived by passing sample *x* through the feature extractor $f_{\phi }$(_▪_). Figure 3 illustrates the structure of the feature extractor, and Table 1 details its parameters, including FC_1, an orthogonal decomposition layer that produces orthogonal features. Additionally, FC_2 is introduced to align with the feature size post skip connection.

#### 2.2.2. Orthogonal Decomposition

To mitigate the impact of redundant information in industrial defect images, orthogonal decomposition is employed for feature processing. Specifically, following ResNet18, a fully connected layer FC_1 is introduced. As demonstrated in Equation (1), the projection of X onto the fully connected layer yields $A^{\prime }$. Notably, feature $A^{\prime }$ at this stage exhibits non-orthogonality, featuring strong correlations and substantial redundant information.

(1) $$A^{\prime }=WX,$$

where *W* is fully connected layer FC_1’s parameters. To reduce the influence of redundant information on the model performance, the parameters of the fully connected layer FC_1 are subjected to SVD in this paper, as depicted in Equation (2). SVD is a matrix decomposition technique that has widespread applications in data analysis and machine learning. SVD can achieve data dimensionality reduction by retaining the main singular values and their corresponding singular vectors. This helps eliminate redundant information, reduce computational complexity, and preserve the key features of the data.

(2) $$W=USV^{T},$$

where *S* is a diagonal matrix, *U* and *V* comprise a unitary matrix. Since the columns of the unitary matrix *U* are orthogonal, after left-multiplying by a diagonal matrix *S*, the resulting matrix $W^{\prime }$’s columns still maintain orthogonality, as shown in Equation (3).

(3) $$W^{\prime }=US$$

**Figure 4:**
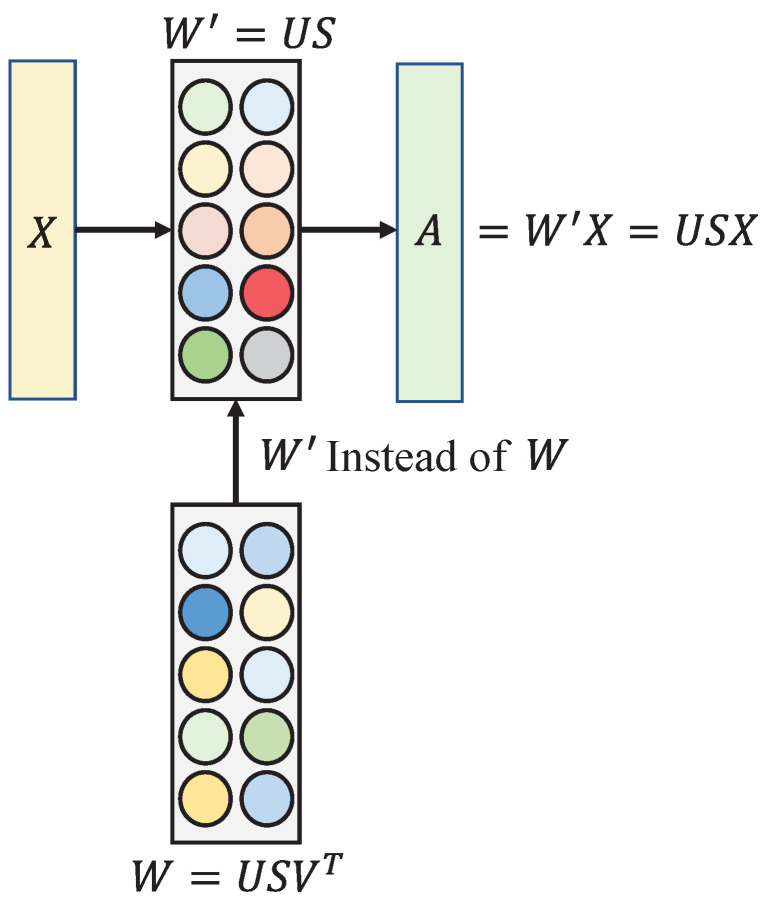
Orthogonal decomposition process.

$W^{\prime }$ becomes an orthogonal matrix, replacing the weight of the fully connected layer with *W*. Equation (4) demonstrates that the projection of feature *X* onto the fully connected layer FC_1 $W^{\prime }$ results in orthogonal feature *A*, which helps diminish the impact of redundant information on the model. To visually illustrate the orthogonal decomposition process, Figure 4 is included in this paper.

(4) $$A=W^{\prime }X=USX$$

However, excessive orthogonal decomposition may eliminate useful correlation information. To address this concern, as illustrated in Figure 2 and Figure 3, this study introduces a skip connection to reintroduce some correlations into the final feature $A_{final}$. Due to the inconsistent feature sizes between the output of ResNet18 and the orthogonal decomposition layer, direct addition by skip connection is not feasible. To address this issue, this paper introduces a fully connected layer into the hopping process. Equation (5) outlines the operation of the skip connection.

(5) $$A_{final}=A+X,$$

where *A* is the orthogonal feature, and *X* is the non-orthogonal feature.

To facilitate a comprehensive understanding of the method proposed in this paper, Algorithm 1 delineates the specific steps involved in orthogonal decomposition and the skip connection operation. For more specific parameter settings, refer to the experimental section.
**Algorithm 1:** Define sample *x* using ResNet18 to obtain *X*. Two fully connected layers: FC_1 $f_{W}$(_▪_) and FC_2 $f_{\theta }$(_▪_). $A_{final}$ is the feature obtained from *x* after undergoing the operations of orthogonal decomposition and skip connection.**Input:** $f_{Re}\left(x\right)$**output:** $A_{final}$*step*1: SVD: $W=USV^{T}$*step*2: Replacing the FC_1 parameter *W* with $W^{\prime }$:$W^{\prime }=US$*step*3: $A_{final}=f_{W^{\prime }}\left(X\right)+f_{\theta }\left(X\right)$

The integration of the orthogonal decomposition operation and the skip connection enables the model to autonomously discern between valuable and redundant information during the training phase. This approach effectively leverages the limited sample data to mitigate the detrimental impact of redundant information on the model performance.

#### 2.2.3. Classifier

Real-time performance is a crucial metric for industrial defect classification models. To enhance the model efficiency, the Euclidean distance is employed as the classifier due to its parameter-free nature and adaptability to data distributions. Additionally, the Euclidean distance represents a specific instance of the Mahalanobis distance under orthogonal inputs. While the Euclidean distance necessitates orthogonal inputs, its strong generalization typically overlooks this requirement in practical applications. Although the final extracted features in this study are not strictly orthogonal, they undergo orthogonalization during feature extraction, which enhances their alignment with Euclidean distance characteristics and contributes to improved classifier performance.

As depicted in Equation (6), the mean of samples $x^{i}$ belonging to the $K^{th}$ class in the support set is computed as the centroid of the $K^{th}$ class in the metric space, referred to as the prototype $c_{K}$.

(6) $$c_{K}=\frac{1}{N}\sum_{1}^{N}f_{\phi }\left(x^{i}\right),$$

where *N* is the number of $K^{th}$ class samples in the support set. The Euclidean distance *d* for query sample $\overset{⌢}{x}\;$ and class prototypes $c_{K}$ is calculated as shown in Equation (7). The probability distribution based on Softmax in metric space is shown in Equation (8).

(7) $$d\left(c_{K},\overset{⌢}{x}\;\right)={∥c_{K}-f_{\phi }\left(\overset{⌢}{x}\;\right)∥}_{2}$$

(8) $$p_{\phi }\left(y=k\left|\overset{⌢}{x}\right.\right)=\frac{\exp\left(-d\left(f_{\phi }\left(\overset{⌢}{x}\right),c_{k}\right)\right)}{\sum_{k^{\prime }}\exp\left(-d\left(f_{\phi }\left(\overset{⌢}{x}\right),c_{k^{\prime }}\right)\right)}$$

The label of the class prototype with the largest probability is the predicted label of the query sample.

ODNet utilizes Log loss with Adam for iterative updates, as illustrated in Equation (9). The model incorporates an L2 regularizer to constrain the parameter space and expedite convergence.

(9) $$\min\;J\left(\phi \right)=-\log p_{\phi }\left(y=k\left|x\right.\right)+\lambda {∥\phi ∥}_{2},$$

where $\lambda {∥\phi ∥}_{2}\;$ represents the L2-regularizer, and λ represents the regularized constant.

**Figure 5:**
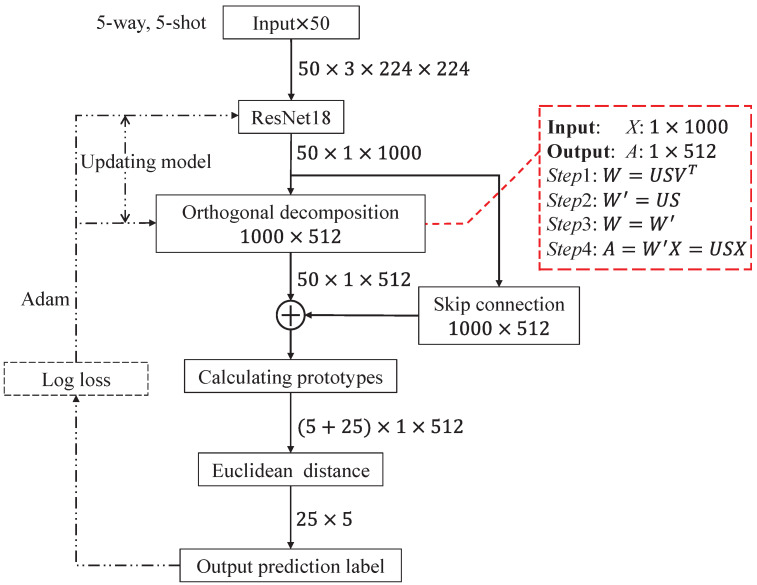
Data flow of ODNet in case of the 5-way, 5-shot. The black dotted line indicates the training stage, and the red dotted line represents the concrete steps of the orthogonal decomposition operation.

Based on the aforementioned model and loss function, Algorithm 2 outlines the procedure. Additionally, to enhance the comprehension of ODNet’s data flow, this study illustrates the data flow encompassing both training and testing phases in Figure 5.
**Algorithm 2:** For an episode, $N_{C}$ is the number of all classes including the support set and query set, $N_{S}$ is the number of samples in each class of the support set, $N_{Q}$ is the number of samples of each class in the query set, $S_{K}$ is the set of  *K*-th samples in the support set, $Q_{K}$ is the query samples collection, and *J* is the loss function. $f_{\phi }\left(\cdot \right)$ denotes the feature extractor, and *d* denotes the Euclidean distance.**Input:**    Training set $D_{tr}=\left\{\left(x_{1},y_{1}\right),\cdots ,\left(x_{N},y_{N}\right)\right\},y_{i}\in \left\{1,\cdots ,K\right\}$, where $x_{i}$ denotes the $i^{th}$ example feature, $y_{i}$ denotes the example $x_{i}$ label, and $\hat{x}$ denotes an example of the query set.**output:** *J**step*1: Class Prototype:$\;\;\;\;C_{K}={\left(N_{S}\right)}^{-1}\times \sum_{\left(x_{i},y_{i}\right)\in S_{K}}f_{\phi }\left(x_{i}\right)$*step*2: Initialization: J←0*step*3: $for\;k\;in\;\left\{1,\cdots ,N_{C}\right\}\;do$*step*4: $\;\;for\;\left(\hat{x},y\right)\;in\;Q_{K}\;do$*step*5:    $J\leftarrow J+d\left(f_{\phi }\left(\hat{x}\right),c_{k}\right)\times {\left(N_{C}N_{Q}\right)}^{-1}+\log\sum_{k^{\prime }}\exp\left(-d\left(f_{\phi }\left(\hat{x}\right),c_{k^{\prime }}\right)\right)$*step*6: endfor*step*7: endfor

To comprehensively verify the performance of the proposed model, we conducted an extensive array of experiments. In addition to accuracy and time assessments, various experiments were performed to evaluate the influence of different classifiers, feature extractors, modules, and parameters on the model’s performance.

## 3. Experiment

This section describes the multiple experiments conducted to validate the proposed model’s performance. Section 3.1 details the datasets utilized and provides the experimental specifics. Section 3.2 presents results showcasing intra-domain and cross-domain accuracy. Section 3.3 outlines a series of ablation experiments examining the influence of various modules or parameters.

### 3.1. Dataset and Implementation

**Figure 6:**
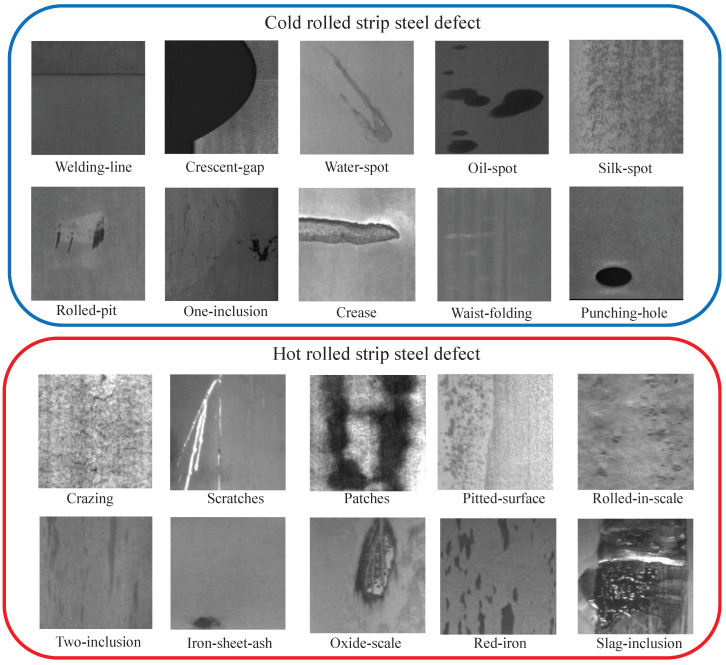
Examples for each class in the FSC-20. The classes in the blue box are cold-rolled defects, and the classes in the red box are hot-rolled defects.

**Dataset**. FSC-20 is a dataset introduced in Song et al. [49] for few-shot strip steel surface defect classification. Figure 6 displays a partial sample of this dataset, which comprises 10 hot-rolled defects (6 types are sourced from the NEU-CLS dataset [50] and 4 types are sourced from the X-SDD dataset [51]) and 10 cold-rolled defects (sourced from the GC10-DET dataset [52]), each with 50 samples. All images were resized to 224×224 pixels. This study enhanced the dataset diversity by rotating the samples several times by 90 degrees. As outlined in Table 2, this work followed Song et al.’s methodology to partition the dataset into training, testing, and validation sets based on class.

**Implementation**. The experiments were conducted in the same setting and environment, which adopted the Microsoft 10 Pro 64-bit Operating System built on a server that applied an Intel(R)Core(TM)i9-10900K with a frequency of 3.70 GHz and a NVIDIA GeForce RTX 3070Ti. The code was written in python3.6. The hyperparameter settings are shown in Table 3.

### 3.2. Precision

In this section, intra-domain and cross-domain experiments are described, including the verification of the real-time performance of the model and a comparison with other methods.

#### 3.2.1. Intra-Domain Results

The experimental results of ODNet on the FSC-20 dataset are presented in Table 4, with the optimal result highlighted in red, the second best in blue, and the third best in green. It is observed that the proposed method outperformed others in both the 5-way 1-shot and 5-way 5-shot scenarios. Specifically, in the 1-shot case, ODNet achieved an accuracy 1% higher than LaplacianShot [53], while in the 5-shot case, it achieved a 5% higher accuracy. These findings demonstrate that the proposed model exhibits strong classification performance on intra-domain tasks and effective discrimination against untrained categories.

#### 3.2.2. Cross-Domain Results

According to the different temperatures, the rolling process of strip steel is categorized into hot-rolled and cold-rolled. As illustrated in Figure 6, the surface defects corresponding to hot-rolled and cold-rolled processes are distinctly different. Hot-rolled defects are often irregular, while cold-rolled defects typically manifest as points or lines. In the intra-domain experiments, the dataset partition did not entirely isolate these two types of defects; instead, both hot-rolled and cold-rolled defects were jointly used for training. Because of the irregularity of hot-rolled defects, its application in testing increases the classification difficulty of the model and can better evaluate the generality of the model. Therefore, this work trained the model on the cold-rolled defects and verified the hot-rolled defects.

Table 5 displays the results of cross-domain experiments, highlighting the optimal, suboptimal, and third-best outcomes in red, blue, and green, respectively. It is evident that the proposed model performed well in both scenarios. Specifically, in the 1-shot case, ODNet achieved an accuracy 3% higher than GTNet [59]. In the 5-shot case, ODNet’s accuracy was slightly lower compared to GTNet [59]. These experimental findings underscore ODNet’s robust generalization ability, demonstrating strong classification performance even across significantly different training and testing categories.

#### 3.2.3. Real-Time Results

In industrial production, aside from accurate defect classification, time is crucial. Timely defect classification enables factories to promptly identify issues and adjust production processes accordingly. To evaluate the real-time performance of the proposed model, this study measured the time taken for an episode (a classification task). As depicted in Table 6, the same color scheme for real-time results as in the preceding section was utilized. It is observed that ODNet achieved suboptimal results in both cases, outperforming the majority of methods. These findings highlight ODNet’s high real-time performance and its ability to swiftly classify defects.

**Figure 7:**
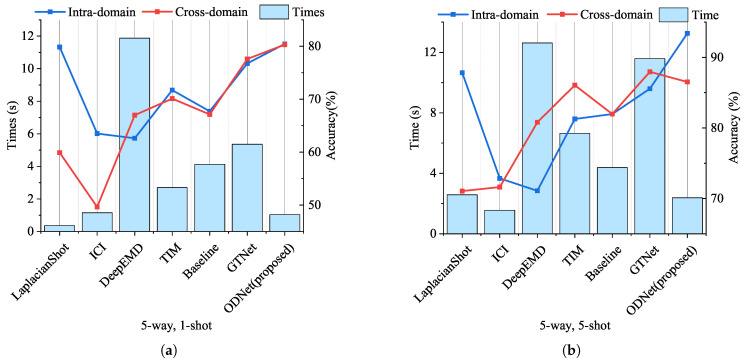
The double *Y*-axis histogram–line chart of the model with real time(s) and accuracy(%). The histogram represents the time it takes for the model to run an episode corresponding to the left *Y*-axis. The blue and red dots in the line chart represent the intra-domain and cross-domain accuracy of the model corresponding to the right *Y*-axis, respectively. (**a**), The real time and accuracy of the model in the case of 5-way, 1-shot. (**b**), The real time and accuracy of the model in the case of 5-way 5-shot.

To visualize the performance of the proposed model, Figure 7 shows the intra-domain accuracy, cross-domain accuracy, and real-time performance. The figure demonstrates that ODNet excelled in both precision and real-time performance compared to other methods, establishing it as the optimal choice for addressing the few-shot strip steel surface defect classification problem.

### 3.3. Ablation

To assess the impact of each module on performance, this section describes a series of ablation experiments exploring the influence of the model parameters and included modules. These experiments aim to further elucidate the model’s performance.

#### 3.3.1. Module Results

To investigate the influence of the backbone, orthogonal decomposition operation, and skip connection on the model performance, we conducted ablation experiments on these three modules. The experimental results are presented in Table 7. It is observed that compared to using the backbone alone, integrating the orthogonal decomposition operation significantly enhanced the model performance. Furthermore, the addition of skip connections following orthogonal decomposition further improved the performance substantially. These effects were validated through experiments: orthogonal decomposition effectively mitigated the impact of redundant information while amplifying the role of pertinent data. The skip connection prevented essential information from being filtered out by orthogonal decomposition. The synergy between these components notably enhanced the model’s classification performance.

To further validate the improvement achieved by the proposed method, additional significance tests were conducted. *t*-tests were employed to analyze significant differences between pairwise combinations across three scenarios, as detailed in Table 7. p<0.05 indicates a significant difference between the compared pairs. The results indicate that all combinations had a p<0.0001, highlighting significant variability in each module’s impact on model performance. The performance improvement of the orthogonal decomposition and the skip connection was notably significant.

We also evaluated the performance of ResNet12 and ResNet34 as feature extractors to determine the optimal choice. The experimental results are presented in Table 8. It was observed that ResNet12’s feature extraction capability was insufficient, resulting in decreased model classification performance. ResNet34 did not significantly enhance the classification performance, and its increased network complexity extended the model’s inference time, thereby reducing the real-time performance. Therefore, ODNet adopted ResNet18 as the backbone to achieve a balance between the real-time performance and the precision of the model.

#### 3.3.2. Classifier Results

The orthogonal method proposed in this paper not only eliminates redundant sample information but also satisfies the orthogonality requirements of Euclidean distance on input features. To assess the impact of different classifiers on model performance, this study also evaluated the cosine distance as a classifier. The experimental results are presented in Table 9. It is evident that in both scenarios, the Euclidean distance outperformed the cosine distance, underscoring the beneficial effect of feature orthogonality on the Euclidean distance performance. Significance testing using *t*-tests was also conducted for both Euclidean and cosine distances. The calculations revealed a p<0.0001, indicating a significant improvement in the effectiveness of the Euclidean distance.

#### 3.3.3. *N* and *K* Results

**Figure 8:**
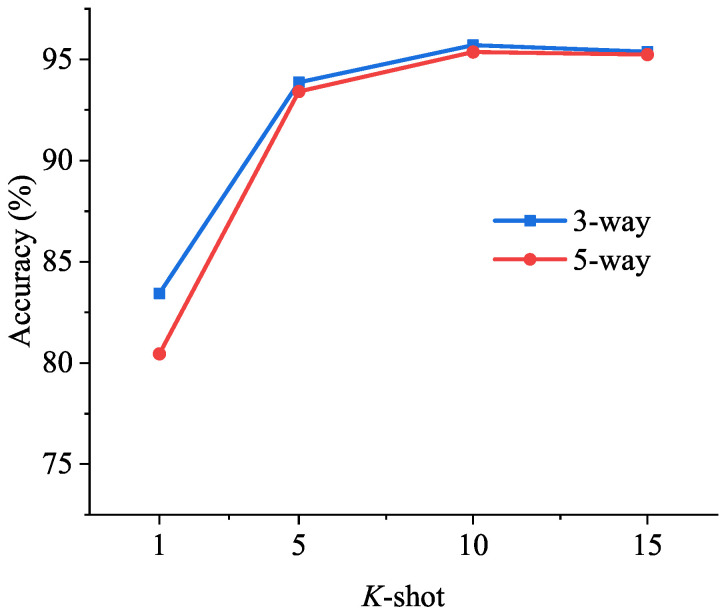
Comparison showing the effect of *N* and *K* for the ODNet. The blue line and red line represent the 3-way and 5-way, respectively. The *X*-axis indicates values of the *K*-shot. The *Y*-axis indicates the test accuracy.

To observe the effect of *N* and *K* on few-shot strip steel surface defect classification, experiments were conducted with $N=\left\{3,5\right\}$ and $K=\left\{1,5,10,15\right\}$, respectively, and the results are shown in Table 10. For a clearer view of the parameter effects on the performance, the line plot depicted in Figure 8 reveals the following insights:

1. With the increase in the number of samples, the model’s performance tends to saturate;
2. The increase in the number of classes increases the classification challenge for the model. However, as the sample number increases, this difficulty becomes negligible.

Therefore, when evaluating model performance, it is appropriate to consider the 5-way 1-shot and 5-way 5-shot scenarios, which reflect the small-scale nature of few-shot learning. The experiments also demonstrate the feasibility of episodes to simulate real-world environments in few-shot strip steel surface defect classification, providing an objective evaluation of model performance.

## 4. Discussion

**Figure 9:**
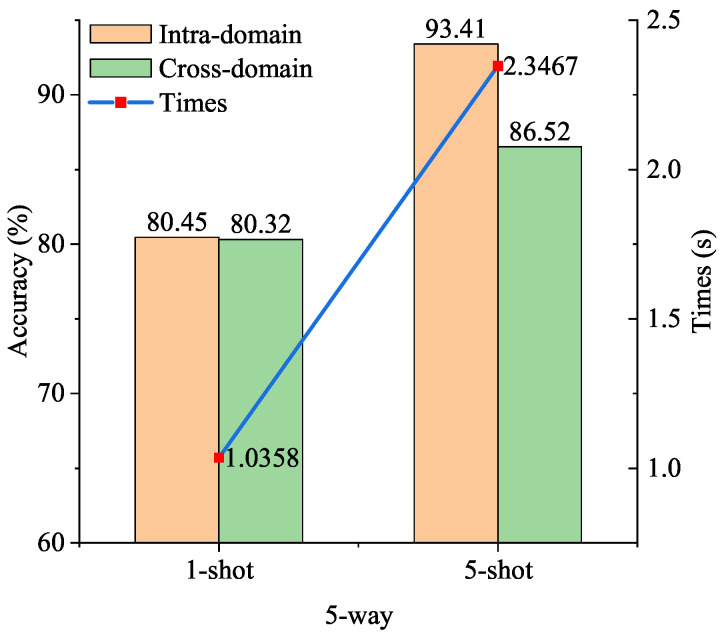
ODNet’s accuracy and real-time statistics.

We verified the performance of ODNet for few-shot strip steel surface defect classification. As depicted in Figure 9, the proposed method exhibits high precision and real-time performance.

In industrial defect samples, redundant information is often prevalent. Valuable information in few-shot learning is limited and precious, and an excess of redundant information can impede the model’s training direction. This interference hinders the model’s ability to effectively discern useful information from redundancy and amplify the importance of the pertinent features. ODNet addresses this issue by subjecting features containing redundant information to orthogonal decomposition. This operation rapidly mitigates the impact of redundant information on the model’s training direction, consequently enhancing the classification performance. Moreover, the skip connection prevents the removal of useful information by the orthogonal decomposition process and fortifies the model’s capacity to distinguish between helpful and redundant information. The efficacy of the skip connection is also evident in the experimental results presented in Table 7. ODNet is a metric-based method, and its orthogonal features partly fulfill the input requirements of Euclidean distance. This enhances the alignment between the feature extractor and the classifier, thus contributing to the performance improvement of the model.

To enhance the real-time performance of the model, this study intentionally simplified its architecture, aiming to achieve improved efficiency. The orthogonal decomposition and skip connection essentially added two fully connected layers. Compared to feature extractors in other mainstream models, this approach significantly reduced the complexity. Additionally, the model employed Euclidean distance as a classifier, which offers stable classification performance without additional parameters. Experimental results, as shown in Table 9, validate the effectiveness of this design in enhancing the real-time capability of the model.

However, as depicted in Figure 9, the cross-domain performance of the model was observed to be slightly lower compared to its intra-domain performance in the 5-shot scenario. Analyzing the reasons, we posit that cross-domain tasks necessitate knowledge transfer, supplemented by prior knowledge introduction. However, ODNet’s orthogonal operation only manages current task knowledge and does not provide prior knowledge to aid learning. Consequently, the proposed model is constrained in its performance on cross-domain tasks.

ODNet theoretically fulfills the requirements of industrial production. In the future, it holds the potential to enable swift and precise detection and classification of surface defects in strip steel on the production line, ensuring product quality aligns with standards, reducing defect rates, and enhancing production efficiency. Nonetheless, its real-world industrial application may encounter challenges, particularly pertaining to the model’s generalization across diverse industrial environments. Addressing this, leveraging techniques such as model pre-training to expedite convergence or employing data augmentation methods to broaden the training dataset could significantly enhance the model’s classification performance in practical industrial settings.

## 5. Conclusions

In this paper, a high real-time orthogonal decomposition network is proposed for few-shot strip steel surface defect classification. ODNet uses SVD to reduce the impact of redundant information on the model. The skip connection can prevent the useful information from being eliminated by the orthogonal operation. Euclidean distance is used as a classifier to limit the overall parameters of the model. The feature with orthogonality is also more in line with the input requirements of Euclidean distance. A large number of experiments show that ODNet has both high precision and high real-time performance, which is more in line with the actual requirements of industrial production. However, compared with intra-domain tasks, the performance of the model in cross-domain tasks needs to be improved. In the future, a priori knowledge can be introduced into the model to assist model training to improve the performance of cross-domain tasks.

## Figures and Tables

**Table 1:** Feature extractor details.

Feature Extractor	Stage	Detail
ResNet18	Pre-processing	Conv2d (5×5, stride 2, pad 1, BatchNorm, RelU)
	Each block	Conv2d (3×3, stride 2, pad 1)
		BatchNorm
		ReLU
		Conv2d (3×3, stride 2, pad 1)
		BatchNorm
		Sum with Input
		ReLU
	Post-processing	AvgPool, Flatten
Orthogonal decomposition layer	Orthogonal decomposition	Fully connected layer (1000×512)
	Skip connection	Fully connected layer (1000×512)
		Sum with Input

**Table 2:** Detailed types of FSC-20.

Training Set (50%) ^[1]^	Validation Set (25%) ^[1]^	Testing Set (25%) ^[1]^
Crescent gap	Welding line	One inclusion
Oil spot	Water spot	Waist folding
Rolled pit	Silk spot	Crazing
Crease	Rolled in scale	Patches
Punching hole	Iron sheet ash	Red iron
Scratches	-	-
Pitted surface	-	-
Two inclusion	-	-
Oxide scale	-	-
Slag inclusions	-	-

^[1]^ The values in parentheses represent the proportion of the number of samples (or classes) of this set to the total number of samples (or classes) of the dataset.

**Table 3:** ODNet’s hyperparameter details.

Training Epochs	Query Sample ^[1]^		Regularizer Constant	Learning Rate	Decay Rate	Decay Episodes	Test Episodes ^[2]^
	Train	Test					
150	5	15	0.5	$10^{-3}$	0.5	2000	1000

^[1]^ The number of query samples used in each episode. ^[2]^ Experiments were used to compute the classification accuracy for models through an average of over 1000 randomly generated episodes from the testing set.

**Table 4:** The intra-domain classification accuracies (%) with 95% confidence intervals on FSC-20. All accuracy results are averaged over 1000 test episodes.

Method	5-Way	
	1-Shot	5-Shot
LaplacianShot [53]	79.86 ± 0.11	87.83 ± 0.08
ICI [54]	63.50 ± 0.66	72.86 ± 0.51
DeepEMD [55]	62.62 ± 0.67	71.10 ± 0.45
Prototypical Nets [56]	43.31 ± 0.34	80.29 ± 0.31
TIM [57]	71.72 ± 0.13	81.27 ± 0.09
Baseline [58]	67.72 ± 0.13	81.97 ± 0.10
GTNet [59]	76.76 ± 0.19	85.56 ± 0.08
ODNet (proposed)	80.45 ± 0.47	93.41 ± 0.26

**Table 5:** The cross-domain classification accuracies (%) with 95% confidence intervals on FSC-20. All accuracy results are averaged over 1000 test episodes.

Method	Cold-Rolled → Hot-Rolled	
	5-Way, 1-Shot	5-Way, 5-Shot
LaplacianShot [53]	59.90 ± 0.19	71.05 ± 0.13
ICI [54]	49.64 ± 0.32	71.62 ± 0.18
Prototypical Networks [56]	49.59 ± 0.37	75.13 ± 0.56
DeepEMD [55]	66.98 ± 0.56	80.80 ± 0.45
TIM [57]	70.11 ± 0.17	86.05 ± 0.08
Baseline [58]	67.12 ± 0.13	81.97 ± 0.10
GTNet [59]	77.61 ± 0.21	87.95 ± 0.08
OdNet (proposed)	80.32 ± 0.22	86.52 ± 0.31

**Table 6:** Test time(s) for an episode.

Method	Time(s)	
	1-Shot	5-Shot
LaplacianShot [53]	0.3581	2.5784
ICI [54]	1.1528	1.5622
DeepEMD [55]	11.8745	12.6195
TIM [57]	2.7006	5.6421
Baseline [58]	4.1243	4.3781
GTNet [59]	5.3617	11.5875
ODNet (ours)	1.0358	2.3467

**Table 7:** Different module classification accuracies (%) with 95% confidence intervals on FSC-20. All accuracy results are averaged over 1000 test episodes.

ResNet18	Orthogonal Decomposition	Skip Connection	5-Way	
			1-Shot	5-Shot
✓			65.27 ± 0.23	81.13 ± 0.34
✓	✓		74.08 ± 0.31	87.65 ± 0.42
✓	✓	✓	80.45 ± 0.47	93.41 ± 0.26

**Table 8:** Different backbone classification accuracies (%) with 95% confidence intervals on FSC-20. All accuracy results are averaged over 1000 test episodes.

Backbone	5-Way	
	1-Shot	5-Shot
ResNet12	77.76 ± 0.62	89.98 ± 0.29
ResNet18	80.45 ± 0.47	93.41 ± 0.26
ResNet34	80.02 ± 0.24	94.35 ± 0.31

**Table 9:** Different classifier classification accuracies (%) with 95% confidence intervals on FSC-20. All accuracy results are averaged over 1000 test episodes.

Classifier	5-Way	
	1-Shot	5-Shot
Euclidean	**80.45 ± 0.47**	**93.41 ± 0.26**
Cosine	48.61 ± 0.42	61.14 ± 0.39

**Table 10:** Different *N* and *K* classification accuracies (%) with 95% confidence intervals on FSC-20. All accuracy results are averaged over 1000 test episodes.

*N*-Way	*K*-Shot	Accuracy
3	1	83.43 ± 0.25
	5	93.87 ± 0.31
	10	95.70 ± 0.42
	15	95.38 ± 0.43
5	1	80.45 ± 0.47
	5	93.41 ± 0.26
	10	95.37 ± 0.29
	15	95.24 ± 0.36

## Data Availability

The data will be made public available on reasonable request.
